# A Comprehensive Analysis of Transcriptomics and Metabolomics Reveals Key Genes Involved in Terpenes Biosynthesis Pathway of *Litsea cubeba* Under Light and Darkness Treatments

**DOI:** 10.3390/ijms26072992

**Published:** 2025-03-25

**Authors:** Jiahui Yang, Yunxiao Zhao, Yicun Chen, Yangdong Wang, Ming Gao

**Affiliations:** 1 State Key Laboratory of Tree Genetics and Breeding, Chinese Academy of Forestry, Beijing 100091, China; yangjiahui0726@163.com (J.Y.); zyx_yunxiao@163.com (Y.Z.); chenyc@caf.ac.cn (Y.C.); wangyangdong@caf.ac.cn (Y.W.); 2 College of Forest, Nanjing Forestry University, Nanjing 210037, China; 3 Research Institute of Subtropical Forestry, Chinese Academy of Forestry, Hangzhou 311400, China

**Keywords:** *Litsea cubeba*, terpenoid, biosynthetic pathway, expression patterns

## Abstract

Light is an important environmental regulator of plant secondary metabolism. Terpenoids, the most abundant secondary metabolites in plants, demonstrate a wide spectrum of biologically significant properties, encompassing antimicrobial, antioxidative, and analgesic activities. *Litsea cubeba* (Lour.) Pers., a core species within the Lauraceae family, exhibits notable pharmacological potential, including antimicrobial and antitumor effects. Here, we found that darkness treatment significantly suppressed terpenoid accumulation in *L. cubeba* fruits. To clarify the molecular mechanisms underlying the regulatory effect of light and darkness treatments on terpenoid biosynthesis, we conducted a comparative transcriptome profiling of *L. cubeba* fruits under light and darkness treatments. A total of 13,074 differentially expressed genes (DEGs) were identified among four sampling time points (L1-L2-L3-L4 vs. D1-D2-D3-D4). These genes were enriched in various pathways, with significant enrichment being observed in the terpenoid and other secondary metabolism pathways. Additionally, the enrichment of DEGs in L2 and D2 stages was further studied, and it was found that nine DEGs were significantly enriched in the monoterpene synthesis pathway. The weighted gene co-expression network analysis (WGCNA) showed that alcohol dehydrogenase (*ADH*), a key enzyme in terpenoid synthesis, had the same expression pattern as *WRKY* and *NAC* transcription factors, suggesting their involvement in the biosynthesis of terpenoids in *L. cubeba*. Expression profiling demonstrated that plastid-localized terpenoid pathway genes were markedly downregulated under darkness treatment. qRT-PCR validation of key genes (*LcDXS3*, *LcHMGS1*, *LcMDS*, and *LcTPS19*) confirmed the reliability of the transcriptome data, with *LcDXS3* exhibiting pronounced declines in expression after 6 h (2.76-fold decrease) and 12 h (2.63-fold decrease) of darkness treatment. These findings provide novel insights into the photoregulatory mechanisms governing terpenoid metabolism in *L. cubeba*.

## 1. Introduction

Terpenoids are the largest class of secondary metabolites found in living organisms, including plants, animals, microbes, and fungi [1,2,3]. Their skeleton is composed of isoprene units, with the number of these units determining the terpenoid type, including monoterpenes (C_10_H_16_), sesquiterpenes (C_15_H_24_), and diterpenes (C_20_H_32_) [4]. Plant terpenoids have dual roles, serving as both essential primary metabolites for growth and precursors for specialized secondary metabolites, such as gibberellins, carotenes, and chlorophylls [5]. Over recent years, terpenoids have attracted considerable attention from the pharmaceutical industry because of their diverse chemical properties, with both terpenoids and their derivatives demonstrating good antibacterial and antioxidant activities. Examples include elemene, a natural compound with anticancer effects [6], as well as α-pinene, paclitaxel, and citral [7]. Notably, taxol in *Taxus brevifolia*, artemisinin in *Artemisia annua*, and ginsenosides from *Panax ginseng* have demonstrated significant potential in disease prevention and treatment [8,9,10]. Monoterpenoids, a prevalent subclass of plant secondary metabolites, have garnered significant attention owing to their multi-functionality, including as antimicrobials and antioxidants, and important chemical components in plant essential oils [11,12]. Environmental factors, including light, which is essential for plant growth and development, are recognized as key regulators of terpenoid biosynthesis. Studies have shown that light induces changes in seedling morphology, participates in seed germination, and triggers flowering [13,14,15,16]. However, the molecular mechanisms underlying the effects of light on secondary metabolism remain unclear. Notably, the light-dependent regulation of fruit-specific metabolites, as exemplified by the industrially valuable monoterpenes (e.g., citral) in *L. cubeba*, remains under investigated.

The biosynthetic pathways of terpenoids in plants have been well characterized, and it is known that monoterpene production primarily occurs through the mevalonate (MVA) and methylerythritol phosphate (MEP) pathways [17,18,19,20]. Terpenoid yield is mainly governed by the efficiency of synthesis of the universal isoprenoid precursors isopentenyl diphosphate (IPP) and dimethylallyl diphosphate (DMAPP). These structural units are converted into terpenoid precursors by key terpenoid biosynthetic enzymes such as terpene synthases (TPS) [1,4]. Understanding the source of terpenoids and clarifying their synthetic pathways and associated regulatory mechanisms can provide strong support for the utilization of terpenoids in food and pharmaceutical industries.

Environmental factors such as light, temperature, drought, and biotic/abiotic stress greatly influence the growth and development of plants, both woody and herbaceous [21,22]. Light plays an essential role in plant secondary metabolic processes, not only providing a source of energy for photosynthesis but also acting as a regulator of plant metabolism and responses to stress [23]. An increasing number of studies have investigated the effects of light on the biosynthesis of plant secondary metabolites, mainly focusing on anthocyanins, sesquiterpene products, artemisinins, and flavonoids [6,24,25]. For instance, in *Solanum melongena* L., light has been shown to modulate anthocyanin accumulation by triggering *SmCRY*-*SmCOP1* interactions, subsequently leading to the suppression of *SmHY5* and *SmMYB1* expression [25]. In ginkgo leaves, it was found that the expression of flavonoid biosynthesis-related genes decreased with decreasing light intensity, accompanied by a reduction in the total flavonoid content [26]. Notably, light intensity directly impacts terpenoid production through the modulation of the MEP pathway [27].

Transcriptome sequencing, a high-throughput DNA sequencing technology, enables the rapid identification of DEGs and the elucidation of physiological processes or molecular mechanisms [28,29]. It also represents a powerful means for analyzing gene expression, predicting gene function, and screening regulatory factors [29,30]. For instance, in *Dendrobium candidum*, transcriptome sequencing was used to elucidate the genes involved in secondary metabolite biosynthesis. Similarly, in *Artemisia annua*, the mechanisms involved in jasmonic acid-induced artemisinin synthesis were deciphered through an integrated transcriptomic analysis. The expression profiles of genes in *Vitis vinifera*, *Fagopyrum tataricum*, *Camellia sinensis*, and *Setaria italica* were also analyzed using transcriptome data, offering valuable insights into the regulation of secondary metabolism [31,32,33,34]. However, the regulatory mechanisms governing terpene biosynthesis in *L. cubeba* fruits under photoperiod variations remain uncharacterized, which limits the utilization of *L. cubeba* fruit.

*Litsea cubeba* (Lour.) Pers. (family: Lauraceae), commonly known as mountain pepper, is recognized as a significant specialty forest tree in China owing to its high essential oil content in roots, stems, leaves, flowers, and fruits [35,36]. The fruit developmental stages of *L. cubeba* are categorized into two distinct phases: the rapid essential oil accumulation phase in mid-July, and the seed maturation phase in September [35,37]. Since the essential oil content in *L. cubeba* fruits is significantly higher than in other plant organs, the essential oil is typically extracted from the fruits. *L. cubeba* essential oil (LCEO) contains abundant terpenoids, particularly monoterpenoids (>90% of the total terpenoid content), including α-pinene, β-pinene, camphene, D-limonene, linalool, and citral. These compounds possess notable biological properties, such as antimicrobial, anti-inflammatory, antioxidant, insecticidal, and mosquito-repellent activities [11,38,39]. *L. cubeba* is a primary source of natural fragrances for the perfume and cosmetic industries. The essential oil can also substitute synthetic chemical preservatives to extend food shelf life. Besides its traditional use as a medicinal plant, *L. cubeba* exhibits diverse therapeutic applications. For instance, mature fruits serve as analgesic agents, decocted roots alleviate rheumatic arthralgia, crushed leaves reduce swelling when applied topically, and fruit residue serves as a natural antibiotic feed additive [40]. Recent studies have further shown that the essential oil of *L. cubeba* has therapeutic potential in the regulation of inflammation and the amelioration of intestinal health [41,42]. Three methods are commonly employed for the extraction of the essential oil of *L. cubeba*—traditional pressing, solvent extraction, and ultrasonic-assisted extraction—with the last method resulting in the highest oil yield [40,43]. With the completion of the high-quality genome assembly, *L. cubeba* was confirmed to have a diploid genome (2*n* = 24), and 31,329 protein-coding genes were identified, which were unevenly distributed across its 24 chromosomes [11]. Additionally, Jiao (2020) et al. sequenced the transcriptomes of various *L. cubeba* tissues (roots, leaves, and flowers), as well as fruits at different developmental stages [44]. The research on the terpenoid synthesis of *L. cubeba* is mainly based on the analysis of the function of a single key gene, including the functional characterization of *LcTPS42* and *LcTPS32*, as well as identifying transcription factors (*LcMYC2*, *LcERF19*, *LcWRKY17*, and *LcMYB106*), which promotes the understanding of the regulation of terpenoid biosynthesis [45,46,47,48]. These studies provide a foundational basis for understanding the molecular mechanisms of terpenoid synthesis in *L. cubeba*. However, how environmental factors (such as light) affect the terpenoid synthesis of *L. cubeba* is still unknown, and it is impossible to analyze the gene expression in *L. cubeba* under light and darkness treatments from the transcription level, which limits the development of the *L. cubeba* industry. Increasing the content of terpenoids in *L. cubeba* is helpful to enhance the potential of *L. cubeba* in the pharmaceutical industry and promote the development of the perfume industry. In conclusion, elucidating the molecular mechanisms underlying terpenoid biosynthesis in *L. cubeba* could directly enhance the plant’s economic value through targeted genetic improvements.

Monoterpenoids and sesquiterpenoids are the main components of *L. cubeba* essential oil, and their synthesis is regulated by both light and darkness. Light can provide energy and carbon sources for terpenoid synthesis through photosynthesis, and can also activate hormone signaling pathways, thereby inducing terpenoid synthesis. Zhao et al. (2020) additionally demonstrated that light exposure and methyl jasmonate (MeJA) both influence monoterpenoid precursor biosynthesis [39]. However, the mechanisms underlying light-mediated regulation of terpenoid biosynthesis remain unclear. In this study, we applied light and darkness treatments to *L. cubeba* fruits. Fruits at the fruit maturation stage (the peak of essential oil synthesis) were selected and subjected to a comparative transcriptomic analysis to elucidate the mechanisms underlying terpenoid biosynthesis regulation [11,37,45]. This study provides an important reference for understanding how environmental factors affect terpenoid synthesis in *L. cubeba*.

## 2. Results

### 2.1. Darkness Treatment Resulted in a Significant Decrease in Monoterpenoid Content in L. cubeba Fruits

**Figure 1:**
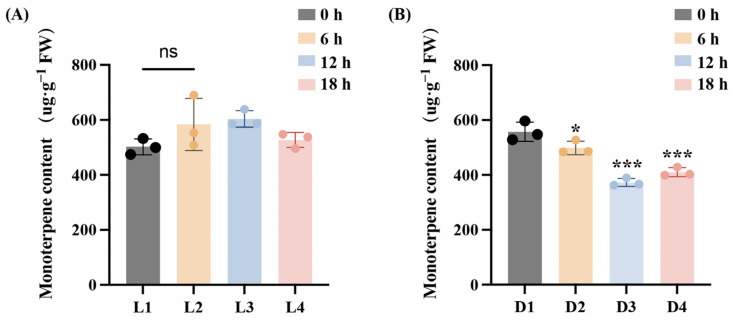
Changes of monoterpene content in *L. cubeba* fruits under light and darkness treatments. (**A**) The monoterpenoid content of *L. cubeba* fruits under light treatment (L1–L4). L2, L3, and L4 are compared with L1. (**B**) The monoterpenoid content of *L. cubeba* fruits under darkness treatment (D1–D4). D2, D3, and D4 are compared with D1. All data are expressed as means ± SD of three biological replicates (* *p* < 0.05, *** *p* < 0.001, Ns indicates that there is no significant difference.).

Light plays an important role in regulating the growth and development of plants. To investigate terpene content dynamics in *L. cubeba* fruits under light and darkness, volatile organic compounds were analyzed by GC–MS. As shown in Figure 1A, no significant variation in terpenoid content was observed during light exposure (L1–L4), although terpenoid levels were marginally higher at the L3 stage (Light-12 h) than at the L1 stage (Light-0 h) (*p* > 0.05). In contrast, darkness treatment resulted in a significant decline in terpene accumulation. Monoterpene levels declined from an initial 557 μg·g^−1^ at D1 (Dark-0 h) to 498 μg·g^−1^ at D2 (Dark-6 h), reaching a minimum of 372 μg·g^−1^ at D3 (Dark-12 h). The monoterpene content at the D4 stage was 409 μg·g^−1^. (Figure 1B). This demonstrates that light is necessary for terpenoid synthesis in *L. cubeba*.

**Figure 2:**
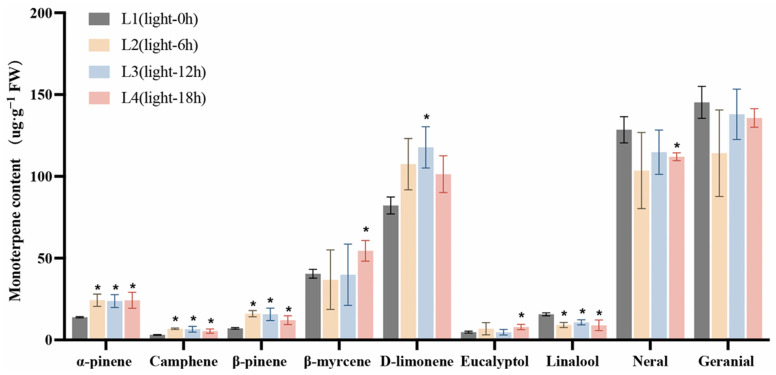
The effects of light treatment (L1–L4) on monoterpenoid content in *L. cubeba* fruits. The contents of α-pinene, camphene, and β-pinene were significantly increased under light treatment, whereas those of linalool, neral, and geranial were decreased at L3 (Light-12 h). L2, L3, and L4 are compared with L1. All data are expressed as means ± SD of three biological replicates (* *p* < 0.05).

Studies have shown that over 90% of *L. cubeba* essential oil consists of monoterpenoids, including α-pinene, camphene, β-pinene, β-myrcene, D-limonene, eucalyptol, linalool, neral, and geranial [11]. Here, we further analyzed the changes in the contents of these important monoterpenoids under light and darkness treatments. The retention times, chemical formulae, and changes in the contents of each compound in the L1 (Light-0 h) to L4 (Light-18 h) stages and the D1 (Dark-0 h) to D4 (Dark-18 h) stages are summarized in Tables S1 and S2, respectively. As shown in Figure 2, the content of each compound at the L2 (Light-6 h), L3 (Light-12 h), and L4 stages was compared with that at the L1 stage. Under light treatment, the contents of α-pinene, camphene, and linalool were significantly increased in the L2 and L3 stages compared with those in the L1 stage. Specifically, the α-pinene content increased from 13.89 µg·g^−1^ at L1 to 24.31 µg·g^−1^ at L2 and 23.83 µg·g^−1^ at L3. The content of camphene increased from 3.09 µg·g^−1^ at L1 to 6.85 µg·g^−1^ at L2, while that of β-pinene rose from 7.16 µg·g^−1^ at L1 to 16.12 µg·g^−1^ at L2, and then slightly decreased to 15.72 µg·g^−1^ at L3. Meanwhile, the contents of β-myrcene, eucalyptol, neral, and geranial showed no significant changes.

**Figure 3:**
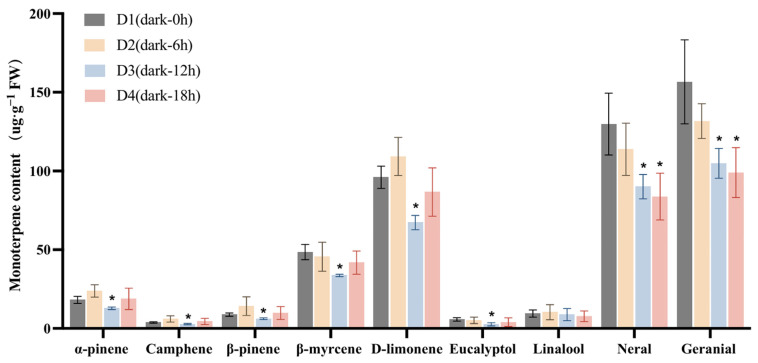
The effects of darkness treatment (D1–D4) on monoterpenoid content in *L. cubeba* fruits. Except for linalool, the contents of other monoterpenoids were significantly decreased at the D3 stage (Dark-12 h), whereas those of neral and geranial showed a further decrease at the D4 stage (Dark-18 h). D2, D3, and D4 are compared with D1. All data are expressed as means ± SD of three biological replicates (* *p* < 0.05).

We then investigated the changes in the contents of these monoterpenoids under darkness treatment (Figure 3). The content of each compound at the D2 (Dark-6 h), D3 (Dark-12 h), and D4 stages was compared with that at the D1 stage. The lowest content of α-pinene was recorded at the D3 stage, decreasing from 18.15 µg·g^−1^ (at D1) to 12.81 µg·g^−1^, while that of β-myrcene decreased from 48.53 µg·g^−1^ at D1 to 33.69 µg g^−1^ at D3. The D-limonene content also decreased, dropping from an initial 96.01 µg·g^−1^ at D1 to 67.30 µg·g^−1^ at D3. Additionally, citral content exhibited a significant reduction compared with D1. Finally, the content of neral decreased from 129.81 to 90.10 µg·g^−1^, and that of geranial declined from 156.61 to 104.86 µg·g^−1^ following 12 h of darkness treatment (D1 to D3). These results further confirmed that darkness treatment significantly reduced the accumulation of monoterpenoids in *L. cubeba* fruits.

### 2.2. Quality Analysis of Transcriptome Sequencing Data Under Light and Darkness Treatments

The ratio of mapped reads to clean reads (alignment rate) serves as a key indicator of transcriptome data quality. In this study, the alignment rate was used to evaluate whether transcriptome sequencing data (NCBI BioProject PRJNA1232969) aligned to the *L. cubeba* reference genome met the requirements for downstream analyses [11]. Table 1 summarizes the alignment results for representative samples under light and darkness treatments, including total reads, total mapped reads, uniquely mapped reads, multi-mapped reads, and the proportion of mapped reads to the sense and antisense strands of the reference genome. The alignment rate ranged from 86.40% at L2 (Light-6 h) to 89.14% at D2 (Dark-6 h). The percentage of mapped reads per sample ranged from 28.91% (L1-1) to 81.44% (D2–3) (Table S3). These results demonstrated that the transcriptome sequencing data were reliable and could be used for subsequent analyses.

### 2.3. Sequencing and Assembly

To investigate the effects of light and darkness treatments on monoterpene synthesis at the transcriptional level, two mRNA libraries were constructed for transcriptome sequencing, comprising a total of 24 samples. After quality filtering, clean data volumes ranged from 6.66 Gb (D3) to 8.97 Gb (D4), with an average error rate of 0.01%. In addition, the Q20 of all the libraries exceeded 98.78% (L4), while the Q30 surpassed 96.7% (L4). The percentage GC content was approximately 46% (Table 2). These results confirmed the high quality of the transcriptome data (Table S4).

**Figure 4:**
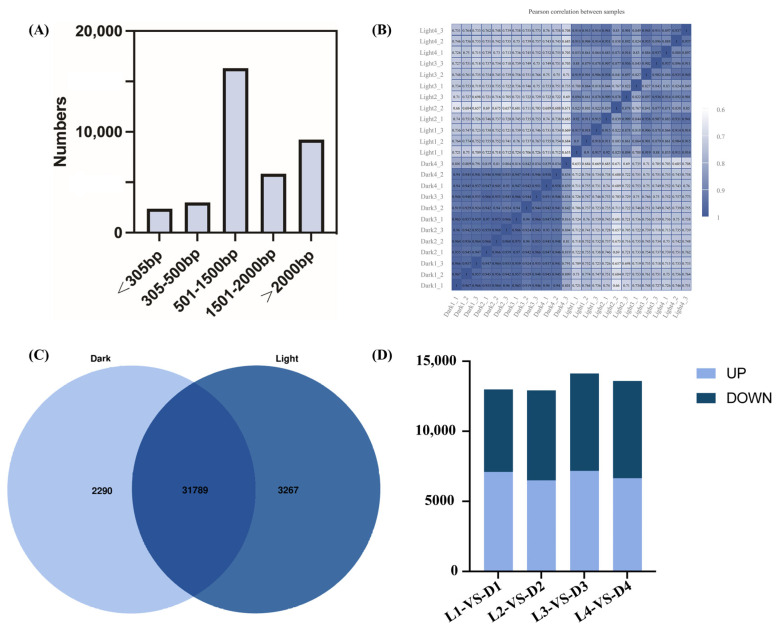
Transcript length distribution, sample correlations, and differential gene expression in *L. cubeba* under light and darkness treatments. (**A**) Transcripts were categorized into five groups of differing bp lengths based on alignment to the reference genome: <305 bp, 305–500 bp, 501–1500 bp, 1501–2000 bp, and >2000 bp. (**B**) Correlation analysis of 24 samples under light and darkness treatments. Higher correlation coefficients indicate greater inter-sample consistency and sequencing quality. (**C**) Comparison of the number of repetitive genes in the transcriptome. Overlapping regions represent shared genes. In total, 2290 and 3267 genes were uniquely expressed under darkness and light treatments, respectively. (**D**) The numbers of DEGs between different samples. The DEGs between the L1 (Light-0 h) and D1 (Dark-0 h) stages; the L2 (Light-6 h) and D2 (Dark-6 h) stages; the L3 (Light-12 h) and D3 (Dark-12 h) stages; and the L4 (Light-18 h) and D4 (Dark-18 h) stages. Up: significantly upregulated, down: significantly downregulated.

Through RNA sequencing and analysis, a total of 41,822 genes were detected. Among them, a total of 37,721 genes were expressed in the transcriptome, with the transcript length distribution shown in Figure 4. Genes with lengths of 501–1500 bp accounted for the largest proportion of the transcriptome (16,304; 43%), followed by those with lengths greater than 2000 bp (9231; 24%) (Figure 4A). The correlation coefficient of the 24 samples from light and darkness treatments is shown in Figure 4B. The higher the correlation coefficient, the better the quality between samples. The overlapping area shows the number of genes common to both light and darkness samples (Figure 4C). Differential expression analysis was performed using DEG2seq with a false discovery rate threshold adjusted cutoff of <0.05 and a fold change cutoff of |log2(FoldChange)| ≥ 1. Figure 4D presents the numbers of upregulated and downregulated DEGs across the four sampling times (L1 vs. D1, L2 vs. D2, L3 vs. D3, and L4 vs. D4) under light and darkness treatments.

### 2.4. Analysis of DEGs

To comprehensively assess the effects of light and darkness treatments on gene expression, we analyzed gene expression levels based on Fragments Per Kilobase of transcript per Million mapped reads (FPKM) values. Among the 37,791 assembled transcripts, 3771 had FPKM values of 0, indicating that approximately 10% of genes were not expressed under darkness treatment. The largest group (12,941 genes; 33% of the total) exhibited FPKM values below 1, suggesting that one-third of the genes were expressed at extremely low levels under darkness treatment and that these genes may inhibit the synthesis of terpenoids. In total, 5085 (13%) and 6428 (17%) transcripts had FPKM values of 1–3 and 3–10, respectively, while 1857 transcripts displayed FPKM values greater than 60, accounting for approximately 4% of the total number of genes.

**Figure 5:**
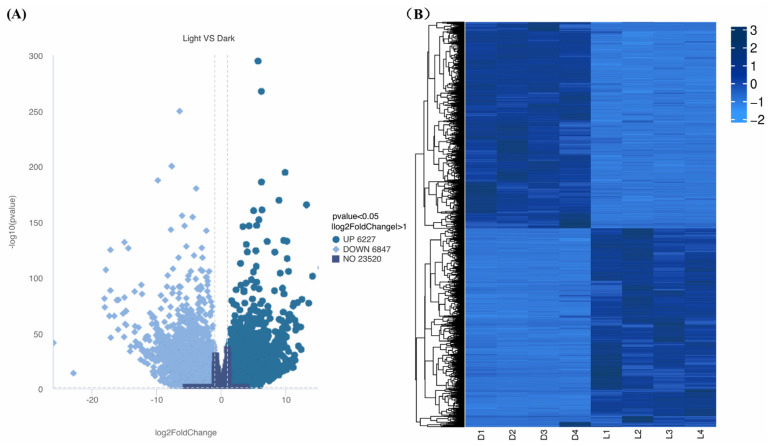
Differential gene expression profiles. (**A**) Upregulated (dark blue) and downregulated (sky blue) DEGs between light and darkness treatments (L1-L2-L3-L4 vs. D1-D2-D3-D4). The horizontal dashed line indicates the significance threshold (a *p*-value of <0.05). (**B**) Hierarchical clustering of the DEGs between light and darkness treatments (L1–L4 and D1–D4).

A total of 13,074 of the genes were found to be differentially expressed between light and darkness treatments (L1-L2-L3-L4 vs. D1-D2-D3-D4), including 6227 that were upregulated and 6847 that were downregulated. Hierarchical clustering analysis was performed based on gene expression patterns (Figure 5A). Under light treatment, most genes in *L. cubeba* showed high expression levels, while under darkness treatment, some genes showed low expression levels (Figure 5B). These results demonstrated that light is essential for terpenoid synthesis in *L. cubeba*.

### 2.5. Functional Analysis of DEGs

Gene Ontology (GO) enrichment analysis of the DEGs (L1-L2-L3-L4 vs. D1-D2-D3-D4) allows us to better understand the functions of genes. This analysis, based on all reference genes, categorizes functions into three categories (Figure S1). Kyoto Encyclopedia of Genes and Genomes (KEGG) pathway enrichment analysis of the DEGs (L1-L2-L3-L4 vs. D1-D2-D3-D4) between light and darkness treatments showed that the DEGs were enriched in five aspects, namely, metabolism, genetic information processing, environmental information processing, cell processes, and organismal systems. The largest proportion of these DEGs was associated with metabolism, which included eleven sub-categories. In total, 136 genes were enriched in the metabolism of terpenoids and 185 were enriched in the biosynthesis of other secondary metabolites (Figure S1).

**Figure 6:**
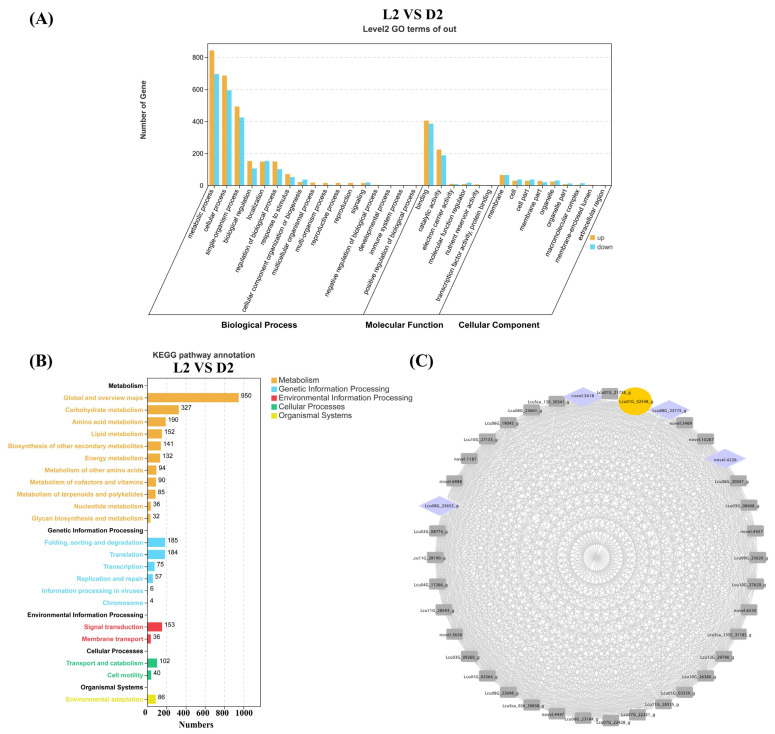
GO, KEGG, and WGCNA analysis of the DEGs (L2 vs. D2). (**A**) GO enrichment analysis of the DEGs (L2 vs. D2) was categorized into biological process, molecular function, and cellular component. (**B**) The DEGs (L2 vs. D2) were enriched in five KEGG metabolic pathway categories. Different colors represent different categories, and the numbers after the pathways represent the number of single genes in the different categories. (**C**) Construction of regulatory networks of TFs and structure genes related to terpenoid biosynthesis. Yellow represents the key enzyme ADH in the terpenoid synthesis pathway, and purple represents transcription factors.

Terpenoid content analysis revealed a significant decrease in *L. cubeba* monoterpenoid content during the D2 stage (Dark-6 h). To further identify key genes involved in terpenoid synthesis during the L2 and D2 stages, we performed GO and KEGG enrichment analyses on all DEGs from the L2 vs. D2 comparison. The GO analysis demonstrated that DEGs in L2 vs. D2 were significantly enriched in metabolic processes, comprising 843 upregulated genes and 696 downregulated genes. This was followed by enrichment in cellular processes, including 686 upregulated genes and 594 downregulated genes. Within the biological process category, only four pathways exhibited more downregulated than upregulated genes: localization, cellular component organization or biogenesis, immune system process, and positive regulation of biological process (Figure 6A). KEGG enrichment analysis revealed that 950 DEGs were collectively enriched in the global and overview maps pathway. The biosynthesis of carbohydrate metabolism pathway showed an enrichment of 327 DEGs. The amino acid metabolism pathway contained 190 enriched DEGs. These three pathways had the most abundant genes. Subsequent analysis identified 85 DEGs enriched in the metabolism of terpenoid and polyketides pathway. Among these, nine genes were closely associated with monoterpene synthesis, including six terpene synthases (*Lcu09G_26017_g*; *Lcu05G_15831_g*; *Lcu01G_01644_g*; *Lcu08G_22878_g*; *novel.2952*; *Lcu01G_01640_g*), two short-chain dehydrogenases (*Lcu01G_00353_g and Lcu01G_00332_g*), and one geraniol synthases (*Lcu06G_19648_g*) (Figure 6B). These DEGs are likely to be involved in terpenoid synthesis in *L. cubeba*.

To identify genes closely associated with terpenoid biosynthesis under light and darkness treatments, we screened DEGs based on the L2 vs. D2 comparison. Using the WGCNA method in the R package (R version 4.2.1), including FPKM values from L1–L4 and D1–D4, we constructed a weighted gene co-expression network with a cutoff value >0.5 and visualized the network using Cytoscape (v3.8.0). In Figure 6C, we identified the *Lcu01G_23775* gene as a key ADH (Alcohol Dehydrogenase) terpene synthase gene, which exhibited extremely high correlations with four transcription factors. ADH functions to catalyze the formation of terpene skeletons and subsequent oxidation/reduction modifications, the final step in citral biosynthesis in *L. cubeba*. The four transcription factors belong to the NAC (*novel.2618* and *novel.4226*) and WRKY (*Lcu08G_23653* and *Lcu08G_23775*) gene families, which are widely involved in regulating plant secondary metabolic processes (Figure 6C) [49,50,51].

### 2.6. The Expression Profile of Genes in the Terpenoid Biosynthetic Pathway in Under Light and Darkness Treatments

**Figure 7:**
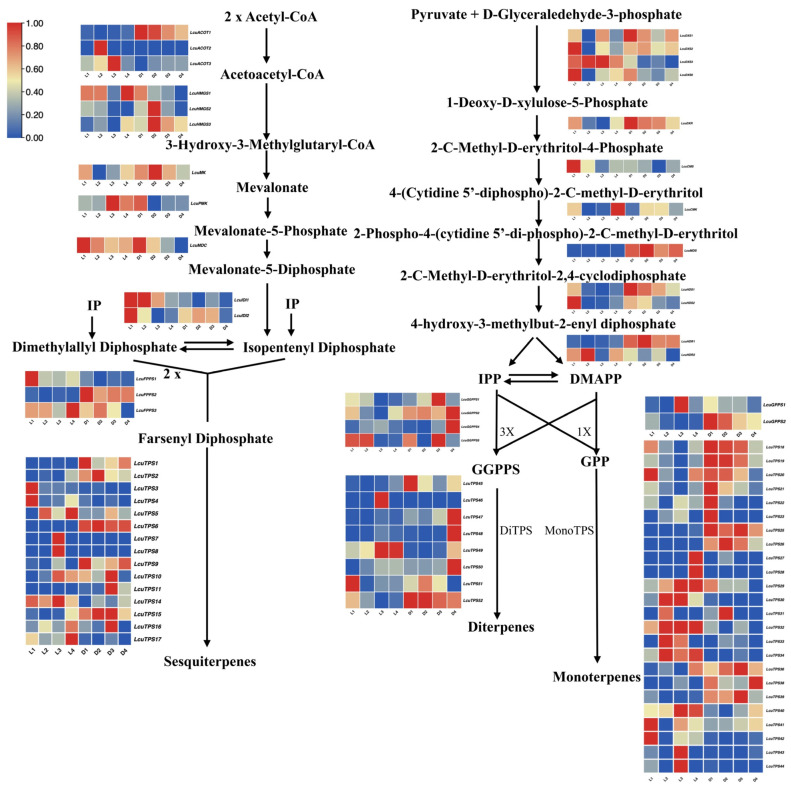
Schematic diagram of the terpenoid biosynthetic pathway in *L. cubeba* under light and darkness treatments (L1–L4 and D1–D4).

To elucidate the changes in the expression of key genes in the terpenoid synthetic pathway in *L. cubeba* under light and darkness treatments, we constructed a schematic diagram of this pathway, incorporating the transcriptomic profiles of critical enzymatic nodes (Figure 7). Within the MVA pathway, key biosynthetic genes (*LcACOT2*, *LcACOT3*, *LcPMK*, *LcIDI1*, *LcTPS5*, *LcTPS14*) exhibited marked transcriptional upregulation under photoperiodic conditions relative to darkness-treated controls. This light-responsive regulatory pattern was similarly observed in the MEP pathway, where *LcDXS1*, *LcDXS3*, *LcHDR2*, *LcTPS21*, *LcTPS23*, *LcTPS27*, *LcTPS28*, *LcTPS29*, *LcTPS30*, *LcTPS31*, *LcTPS32*, *LcTPS33*, and *LcTPS34* demonstrated significant upregulation under light treatment compared to darkness treatment. Conversely, genes such as *LcDXR*, *LcMDS*, *LcHDR1*, *LcGPPS2*, *LcTPS18*, *LcTPS19*, and *LcTPS20* exhibited elevated expression levels in the darkness treatment process. In addition, we found that under the darkness treatment (D3–D4), the expression levels of most genes in the MVA and MEP pathways, including those in the *HMGS*, *MK*, *PMK*, *FPPS*, *DXS*, *DXR*, and *HDS* gene families, were significantly decreased. Notably, the expression levels of *LcACOT1*, *LcHMGS1*, *LcHMGR3*, *LcMDC*, *LcDXS1*, *LcDXS6*, *LcCMS*, *LcHDS1*, *LcHDS2*, and *LcGPPS2* exhibited strong positive correlations with monoterpene accumulation dynamics during darkness treatment (Figure 7). These findings indicates that transcriptome sequencing represents an important method for screening candidate genes that can enhance terpenoid production in breeding programs.

### 2.7. Validation of Some Important DEGs Profiling Using RT-qPCR

**Figure 8:**
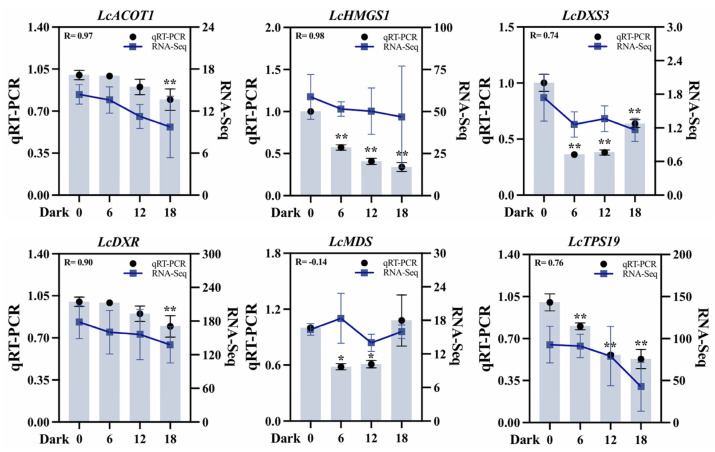
qRT-PCR validation of six differentially expressed key genes across four stages (D1–D4) under darkness treatment. *ACOT* (acyl-CoA thioeaterase), *HMGS* (hydroxymethylglutaryl-CoA synthase), *DXS* (1-deoxy-D-xylulose-5-phosphate synthase), *DXR* (1-deoxy-D-lxylulose 5-phosphate reductoisomerase), *MDS* (2-C-methyl-D-erythritol-2,4-cyclodiphosphate synthase), and *TPS* (terpene synthase). *UBC* was used as an internal reference gene for normalization. D2, D3, and D4 are compared with D1. Student’s *t*-test was used for significance analysis. All data are expressed as means ± SD of three biological replicates (* *p* < 0.05, ** *p* < 0.01).

To validate the transcriptome dataset, six DEGs associated with terpenoid biosynthesis were selected: two from the MVA pathway (*LcACOT1*, *LcHMGS1*), three from the MEP pathway (*LcDXS3*, *LcDXR*, *LcMDS*), and one terpenoid synthase-encoding gene (*LcTPS19*). Quantitative analysis revealed that the expression patterns of most of these genes were highly consistent with the transcriptome data. For instance, *LcACOT1* exhibited a Pearson correlation coefficient of 0.97 with transcriptome data across the four darkness treatment stages (D1–D4), alongside a significant positive association (R = 0.88) with terpene accumulation under darkness treatment. In addition, the correlation between the qRT-PCR results for *LcHMGS1* and *LcDXR* and the transcriptome data reached a value above 0.8 (Figure 8). Discrepancies were observed for *LcMDS*, potentially attributable to differences in sensitivity and specificity between qRT-PCR and sequencing technology. Collectively, qRT-PCR validation substantiated the reliability of the transcriptome data and the derived gene expression profile.

## 3. Discussion

The biosynthesis of terpenoids primarily relies on the MEP pathway (plastids) and MVA pathway (cytosol). Darkness indirectly regulates terpenoid biosynthesis genes by suppressing light-signaling transduction pathways. However, the molecular mechanism of dark-affecting terpenoid synthesis in *L. cubeba* is unknown. To address this knowledge gap, we subjected *L. cubeba* fruits to both light and darkness treatments. Light treatment exhibited no significant impact on the total monoterpenoid content in *L. cubeba* fruits (Figure 1A). However, certain monoterpenoids, including α-pinene, camphene, and β-pinene, showed significant increases following 6 h of light exposure, demonstrating higher light responsiveness compared to other monoterpenoids. (Figure 1B). These results align with findings in mint (*Mentha* spp.) and wild mint (*Mentha arvensis*), indicating that light influences monoterpenoid accumulation [27,52,53]. Given these findings, we next constructed transcriptome sequencing libraries under light and darkness treatments and investigated gene expression changes during terpenoid synthesis in *L. cubeba*.

Transcriptome-based candidate gene screening has proven to be an effective, precise, and robust tool for use in molecular breeding in various plant species, including *Camellia vietnamensis* Huang, *Tanacetum coccineum*, *Dendrobium catenatum*, *Pyrus communis*, *Taxus chinensis*, and *Carthamus tinctorius* L. [54,55,56,57,58]. In this study, we established two RNA-seq libraries and profiled gene expression in *L. cubeba* fruits following light and darkness treatments. Approximately 7 Gb of high-quality reads were generated, yielding 37,791 unigenes. Principal component analysis (PCA) identified strong inter-sample correlations (Figure 4C). Through reference genome alignment, we annotated gene structures (e.g., length, initiation sites) and predicted gene function. Comparative analysis of *L. cubeba* fruit transcriptomes following light and darkness treatments identified 6227 upregulated and 6847 downregulated DEGs, with some DEGs lacking annotations in public databases. These unannotated DEGs may represent *L. cubeba*-specific candidate genes involved in terpenoid biosynthesis, aligning with prior findings [6]. Through KEGG pathway enrichment analysis, which enables functional prediction of genes and the elucidation of their roles within metabolic networks [59], we found that light-responsive DEGs were potentially involved in pathways such as metabolism of terpenoids and polyketides, biosynthesis of other secondary metabolites, and signal transduction (Figure S1), which facilitated the identification of candidate terpenoid biosynthesis-related genes.

Plant terpenoid biosynthesis is predominantly regulated by environmental cues, such as light [60], and plant hormones, such as methyl jasmonate [27,61]. Monoterpenes are usually biosynthesized in plastids, with photosynthesis providing precursors, ATP, and NADPH [62]. Transcriptome analysis revealed that both the total terpenoid content and the levels of key monoterpenoids were significantly reduced under darkness treatment (Figure 1). Notably, darkness treatment significantly suppressed the expression of genes in the MVA and MEP pathways, particularly *LcHMGS1*, *LcPMK*, *LcDXS3*, *LcDXS6*, *LcGGPPS2*, *LcGGPPS4*, and *LcTPS42* [63] (Figure 7). Studies have demonstrated that *LcDXS3* and *LcTPS42* overexpression significantly enhances monoterpene accumulation in *L. cubeba* [11], suggesting that light regulates monoterpene biosynthesis in *L. cubeba* through a dual mechanism—the provision of biosynthetic precursors/cofactors and transcriptional control via light-responsive promoter elements, which is consistent with previous research [39].

In conclusion, this study provides a reference for the effect of light on terpene synthesis and the production of essential oil in *L. cubeba.* Darkness treatment significantly reduced the content of monoterpenes in *L. cubeba* fruits, as well as the expression levels of genes in the terpene synthetic pathway, including *LcACOT1*, *LcHMGS1*, *LcDXR*, *LcDXS3*, and *LcMDC*. The expression of TPS-encoding genes, particularly *LcTPS1*, *LcTPS9*, *LcTPS19*, and *LcTPS22* was also significantly downregulated following darkness treatment. This work elucidated the transcriptional mechanisms underlying terpenoid biosynthesis in *L. cubeba*, and the identified DEGs provide valuable targets for enhancing the production of essential oil in this species through metabolic engineering.

## 4. Materials and Methods

### 4.1. Light and Darkness Treatments of L. cubeba Fruits

*Litsea cubeba* samples were collected from the subtropical forestry research plantation in Fuyang District, Hangzhou City, Zhejiang Province. The stages of *L. cubeba* fruit development have previously been reported, including the fruit growth stage, fruit maturation stage, and fruit ripening stage, with 75 days after full bloom (DAF 75) identified as the peak of essential oil synthesis [11,37,45]. The fruiting branches (50–60 cm) collected in the experimental field at the DAF 75 stage were immediately put into sterile water and transported to the laboratory. Before the light and darkness treatments, the fruiting branches were pre-cultured collectively for 4 h in an illuminated growth chamber (temperature 26 °C, 60% relative humidity) to stabilize the physiological state of the fruits. During the treatment, the fruit was always on the branches and was not stressed.

The light and darkness treatments lasted for 18 h, with samples collected at 0, 6, 12, and 18 h. Throughout the entire treatment period, the physiological state of the detached fruit-bearing branches remained stable, and no evidence of water loss was observed. This method has been used many times in vitro treatment research, and it truly reflects the physiological changes and molecular mechanisms in vivo [39,45,64,65,66,67]. The feasibility and reliability of this method have been recognized. The fruit-bearing branches were randomly divided into two groups: group I received light treatment (maintained under constant illumination in the growth chamber), and group II was subjected to darkness treatment (fully enclosed in light-impermeable black containers within the same chamber). Group I samples collected at 0, 6, 12, and 18 h were named L1, L2, L3, and L4. Group II samples collected at 0, 6, 12, and 18 h were named D1, D2, D3, and D4, yielding a total of 24 samples. These were subsequently submitted to Novogene Co., Ltd. (Beijing, China) for transcriptome sequencing.

### 4.2. Volatile Component Analysis

Volatile Compounds from *L. cubeba* Fruits:

Three biological replicates from the fruit of *L. cubeba* plants (3.0 g) were ground into a uniform powder under liquid nitrogen with a mortar and pestle. The 0.5 g powder was placed in a 20-mL solid-phase microextraction (SPME) vial containing 50-μL aliquot of freshly prepared ethyl decanoate (802180, Merck, Darmstadt, Germany) solution as an internal standard, then sealed and placed in 4 °C until used [39]. Analytical procedures were performed on an Agilent 8890-5977B (Agilent Technologies, La Jolla, CA, USA) GC–MS system and comprised three sequential phases—HS-SPME (Headspace Solid-Phase Microextraction) pre-treatment involving 1 min of equilibration at 50 °C via the CTC system; 50 min of combined extraction/adsorption; and desorption for 5 min at 250 °C in the GC–MS injection port.

Gas chromatography (GC) conditions: DB-5MS (60 m × 0.25 mm ID × 0.25 μm, Agilent) chromatographic column was used, with the injector temperature set to 50 °C for 2 min, ramped up to 80 °C at 3° C/min (2 min hold), increased at 5 °C/min to 180 °C (1 min hold), followed by a 10°C/min rise to 230 °C (5 min hold), then heated at 20 °C/min to 250 °C (3 min hold), and finally elevated at 40 °C/min to 280 °C (5 min hold). The carrier gas was high-purity helium (99.999%), and the column flow rate was 1.5 mL/min.

Mass spectrometer (MS) conditions: Ionization mode: EI, electron energy 70 eV, ion source temperature 230 °C, interface temperature 250 °C, acquisition mode: full scan; mass scan range 50–600 *m*/*z*.

Qualitative analysis of compounds: Volatile compounds were identified by comparing the mass spectra with the published standard substance retaining index as well as information from the National Institute of Standards and Technology (NIST). The specific methods are as follows: The original data acquired by GC–MS were first deconvoluted using Masshunter Agilent Analysis (Agilent) to obtain the Retention time (RT), Compound name, CAS number, Matching factor, Structural formula, and Component area. The single peak was then filtered, and only the peak area data of a single group or all groups with a null value no >50% were retained. Internal standards were used to normalize data processing. Each chromatographic peak area represented the relative content of the corresponding substance. Finally, the integral data of all chromatographic peak areas were exported for statistical analysis. The compounds were determined by computer matching and the retention time of each compound was reported in the literature. For compound quantitative determination, an internal standard normalization method was employed, with compound contents calculated as ethyl decanoate equivalents based on the ratio of individual peak areas to the internal standard peak area.

### 4.3. RNA Extraction and cDNA Library Construction

After sample preparation, total RNA was extracted from each sample using the RNAprep Pure Plant Plus Kit (Tiangen, DP441, Beijing, China) following the manufacturer’s protocol, and RNA quality and concentration were assessed. RNA quality control was performed primarily using the Agilent 2100 Bioanalyzer (Agilent Technologies, La Jolla, CA, USA). After sample detection, the cDNA library was constructed. The library was constructed according to the general method of NEB, including mRNA acquisition, RNA fragmentation, reverse transcription, adapter ligation, and double-stranded cDNA synthesis. The qualified libraries were sequenced on an Illumina platform.

### 4.4. Transcriptome Sequencing

The image data obtained through high-throughput sequencing were transformed into reads using CASAVA base recognition. To ensure the quality of subsequent data analysis, the original data were screened and filtered, and high-quality data with good Q20 and Q30 scores and corrected for GC content were obtained. The published *L. cubeba* genome (GenBank accession: PRJNA1232969) served as the reference genome and annotation source. The paired clean reads were compared with the reference genome using HISAT2 v.2.0.5 software. New transcript prediction was carried out using StringTie v2.2.3 software to splice complete and accurate transcripts and better predict the expression levels of transcripts.

### 4.5. Gene Annotation Analysis

FPKM serves as a standardized metric for quantifying gene expression levels in transcriptome sequencing [68]. The identification of DEGs involves three sequential analytical phases: initial normalization of raw sequencing read counts, subsequent statistical computation of significance probabilities (*p*-values) for expression differences, and multiple hypothesis testing corrections. DEGs between light and darkness treatments (L1-L2-L3-L4 vs. D1-D2-D3-D4) samples were identified using DEG2seq with a false discovery rate threshold adjusted cutoff of <0.05 and a fold change cutoff of |log2(FoldChange)| ≥ 1 (https://magic.novogene.com/, accessed on 24 October 2024). This dual-filter criterion ensures the selection of genes demonstrating at least a 2-fold difference in expression (equivalent to fold change ≥ 2 or ≤0.5) between comparison groups. A comprehensive functional annotation and pathway enrichment analysis of the DEGs was conducted using the GO and KEGG databases.

### 4.6. Weighted Gene Coexpression Network Analysis

WGCNA from the R package was applied to identify genes associated with terpenoid biosynthesis using a weighted cut-off value > 0.50. Subsequently, the co-expression network was constructed and visualized using Cytoscape software (v3.8.0).

### 4.7. The Expression Profile of Genes Involved in the Terpene Biosynthetic Pathway

To further investigate the mechanisms involved in the biosynthesis of terpenoid compounds in *L*. *cubeba* fruits under light and darkness treatments, expression profiling of all the genes involved in terpenoid biosynthetic pathways was undertaken, and comprehensive gene expression profiles associated with the terpenoid biosynthetic pathway were generated, including expression patterns under light (L1–L4) and darkness (D1–D4) treatments. Heat maps were drawn to display the gene expression patterns of the FPKM values using TBtools software v2.210 [69]

### 4.8. qRT-PCR Validation of Differentially Expressed Genes

Target-specific primers were designed with Primer3 (v.0.4.0) software (https://bioinfo.ut.ee/primer3-0.4.0/, accessed on 18 November 2024), and the specificity of each pair of primers was detected using Primer-BLAST at NCBI (https://www.ncbi.nlm.nih.gov/tools/primer-blast/, accessed on 18 November 2024) [29,30]. Reverse transcription was performed with the Takara PrimeScript RT reagent Kit (Takara, Dalian, China) using 1 μg of total RNA per reaction to ensure consistency. qPCR analysis was performed using the TB Green Premix Ex Taq II Fast qPCR Kit (Takara, Dalian, China) in 25-μL reaction volumes containing 12.5 μL of Premix, 1 μL each of forward/reverse primers (10 μM), 2.5 μL of cDNA, and nuclease-free water. Amplification was carried out according to the kit instructions for a total of 40 cycles. Three technical replicates per sample were analyzed, with relative expression levels calculated via the 2^−ΔΔCt^ method [11,39]. *UBC* served as the internal reference gene. The primer sequences are detailed in Supplementary Table S5.

### 4.9. Statistical Analysis

The data were analyzed using IBM SPSS Statistics 25 software and are expressed as means ± standard deviation (SD). *p*-values of ≤0.05 were considered significant. Graphs and tables were created in Excel and GraphPad Prism 9. Student’s *t*-test was used to analyze the significance of differences (* *p* < 0.05, ** *p* < 0.01).

## 5. Conclusions

In this study, the transcriptome data of *L. cubeba* under light and darkness treatments were compared and analyzed in detail, providing a better understanding of the molecular mechanisms involved in terpenoid synthesis in this species. Differential expression analysis showed that 13,074 genes were differentially expressed between light and darkness treatments across four sampling time points (L1-L2-L3-L4 vs. D1-D2-D3-D4). These DEGs were enriched in various pathways, with significant enrichment observed in the terpenoid and other secondary metabolism pathways. Additionally, the expression of terpenoid synthetic pathway genes in plastids was significantly inhibited under darkness treatment. qRT-PCR validation of differential gene expression (particularly *LcDXS3*, *LcHMGS1*, *LcMDS*, and *LcTPS19*) further confirmed the reliability of the transcriptome data. Of these genes, *LcDXS3* showed significant inhibition at 6 h (2.76-fold reduction) and 12 h (2.63-fold reduction) under darkness treatment. These results provide valuable insights into the molecular mechanism underlying terpenoid synthesis in *L. cubeba* fruit, especially from the perspective of environmental factors. Meanwhile, these findings provide a new insight into the light regulation mechanism of terpenoid metabolism in Lauraceae plants.

## Figures and Tables

**Table 1:** Sequence alignment results of sample sequencing data and reference genome.

Treatment	Total Reads	Total Map	Unique Map	Multi Map	Proper Map
L1 (Light-0 h)	50,905,702	88.78	75.63	13.14	68.22
L2 (Light-6 h)	47,260,316	86.40	71.14	15.26	62.72
L3 (Light-12 h)	49,269,064	86.93	71.52	15.42	62.60
L4 (Light-18 h)	49,721,182	86.28	73.65	12.64	64.60
D1 (Dark-0 h)	46,314,858	87.58	79.38	8.20	71.16
D2 (Dark-6 h)	48,571,858	89.14	80.91	8.23	72.70
D3 (Dark-12 h)	48,092,412	88.91	80.59	8.32	72.77
D4 (Dark-18 h)	55,470,416	88.69	80.6	8.08	71.98

Note: Total reads: number of clean reads; Mapped reads: number of reads on *L. cubeba* genome and their proportion in clean reads; Multi map: the number of reads matched to the unique position of genome; Proper map: comprehensive comparison rate.

**Table 2:** Statistics of transcriptome data under light and darkness treatments.

Treatment	Raw_Bases	Clean_Reads	Q20	Q30	GC_pct
L1 (Light-0 h)	7.18 G	44,639,390	98.85	96.9	46.9
L2 (Light-6 h)	7.57 G	47,260,316	98.79	96.82	46.48
L3 (Light-12 h)	6.99 G	42,747,128	98.91	97.03	45.56
L4 (Light-18 h)	7.45 G	46,877,574	98.78	96.7	46.6
D1 (Dark-0 h)	7.36 G	45,436,082	98.85	96.91	45.46
D2 (Dark-6 h)	7.62 G	48,571,858	98.87	96.94	46.1
D3 (Dark-12 h)	6.66 G	42,364,516	98.8	96.75	46.25
D4 (Dark-18 h)	8.97 G	55,470,416	98.84	96.83	45.95

Note: Treatment is the name of the sample; raw_bases is the total base number of original data; clean_reads are the number of remaining reads after filtration; Q20 rate is the proportion of bases with a mass value greater than 20 (error rate less than 1%) in the total sequence after filtration; Q30 rate is the proportion of bases with a mass value greater than 30 (error rate less than 0.1%) in the total sequence after filtration; GC_pct indicates the GC content of the filtered data.

## Data Availability

Data are available upon request.

## Supplementary Materials

The following supporting information can be downloaded at: https://www.mdpi.com/article/10.3390/ijms26072992/s1.
