# Supplementary material for: A Comprehensive Analysis of Transcriptomics and Metabolomics Reveals Key Genes Involved in Terpenes Biosynthesis Pathway of Litsea cubeba Under Light and Darkness Treatments
Source: Int J Mol Sci. 2025 Mar 25;26(7):2992. doi: 10.3390/ijms26072992 (PMC11988597; doi:10.3390/ijms26072992)
Supplement: Supplementary file 1 [file ijms-26-02992-s001.zip › ijms-3496050-supplementary.pdf]

(A) (B)

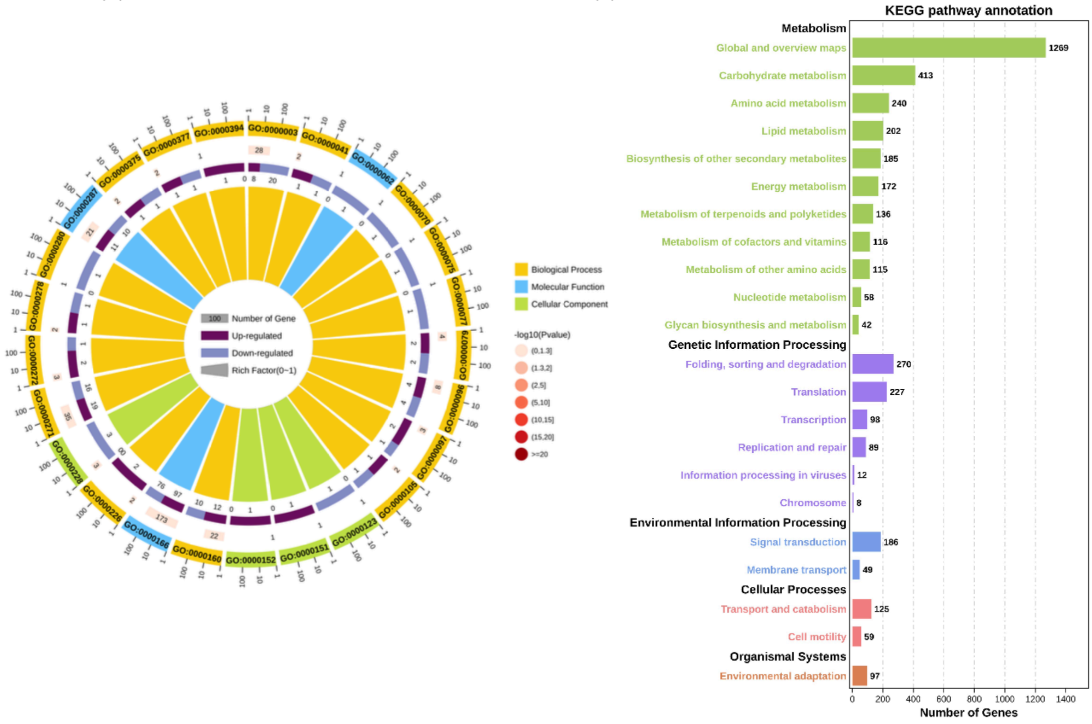

Supplementary Figure S1 Enrichment analysis of GO and KEGG function of differential genes (L1-L2-L3-L4 vs. D1-D2-D3-D4).

Supplementary Table S1 Effect of continuous light treatment on terpenoid importent content in *L. cubeba* fruit.

RT	Terpenoids	Chemical	L1 (Light-0 h)	L2 (Light-6 h)	L3 (Light-12 h)	L4 (Light-18 h)
14.4	α-Pinene	$C_{10}H_{16}$	13.89±0.38	24.31±3.74*	23.83±3.93*	24.23±4.87
15.3	Camphene	$C_{10}H_{16}$	3.09±0.21	6.85±0.48*	6.57±1.69*	5.42±1.33
16.7	β-Pinene	$C_{10}H_{16}$	7.16±0.49	16.12±1.89*	15.72±3.78*	12.14±2.69
18.1	β-Myrcene	$C_{10}H_{16}$	40.49±2.66	36.82±18.18	39.88±18.73	54.50±6.33
19.2	D-Limonene	$C_{10}H_{16}$	82.17±5.18	107.47±15.67	117.73±12.61*	101.36±11.26
19.4	Eucalyptol	$C_{10}H_{18}O$	4.82±0.58	6.88±3.72	4.73±1.68	7.91±1.54
22.1	Linalool	$C_{10}H_{18}O$	15.66±0.88	9.19±1.54*	10.77±1.56*	8.94±3.20
27.3	Neral	$C_{10}H_{16}O$	128.50±8.04	103.57±23.28	114.81±13.54	111.97±2.38
28.2	Geranial	$C_{10}H_{16}O$	145.26±9.74	114.14±26.40	137.95±15.37	135.72±5.66

Supplementary Table S2 Effect of continuous dark treatment on terpenoid importent content in *L. cubeba* fruit.

RT	Terpenoids	Chemical	D1 (Dark-0 h)	D2 (Dark-6 h)	D3 (Dark-12 h)	D4 (Dark-18 h)
14.4	α-Pinene	C10H16	18.15±2.26	23.80±3.95	12.81±0.79*	18.77±6.76
15.3	Camphene	C10H16	3.80±0.42	6.00±1.96	2.75±0.38*	4.43±1.97
16.7	β-Pinene	C10H16	8.77±1.05	14.15±5.88	6.19±0.56*	9.82±4.04
18.1	β-Myrcene	C10H16	48.53±4.84	45.54±9.22	33.69±0.71*	41.80±7.40
19.2	D-Limonene	C10H16	96.01±7.08	109.21±12.12	67.30±4.54*	86.64±15.36
19.4	Eucalyptol	C10H18O	5.73±1.07	5.13±2.09	2.62±1.00*	4.02±2.74
22.1	Linalool	C10H18O	9.46±2.21	10.30±4.79	8.79±3.80	7.71±3.34
27.3	Neral	C10H16O	129.81±19.64	113.76±16.57	90.10±7.72*	83.74±14.87
28.2	Geranial	C10H16O	156.61±26.65	131.68±11.03	104.86±9.45*	98.98±15.82

Note: the symbol "*" indicate that there is a significant increase with 0 h treatment. The unit of each compound is $\mu g \cdot g^{-1}$. All data are the means ± SD from 3 biological replicates (*$P < 0.05$, Student's t-test).

Supplementary Table S3 Summary of sequencing data quality of all samples under light and dark treatment.

**Sample**	**total_reads**	**total_map**	**unique_map**	**multi_map**	**read1_map**	**read2_map**	**positive_map**	**negative_map**	**splice_map**	**unsplice_map**	**proper_map**
D1_1	45436082	39674351(87.32%)	35941482(79.1%)	3732869(8.22%)	18008578(39.63%)	17932904(39.47%)	18009745(39.64%)	17931737(39.47%)	14040441(30.9%)	21901041(48.2%)	31896038(70.2%)
D1_2	43074982	37559255(87.2%)	34069948(79.09%)	3489307(8.1%)	17069365(39.63%)	17000583(39.47%)	17065518(39.62%)	17004430(39.48%)	13105051(30.42%)	20964897(48.67%)	30405288(70.59%)
D1_3	46314858	40562616(87.58%)	36764346(79.38%)	3798270(8.2%)	18436930(39.81%)	18327416(39.57%)	18407303(39.74%)	18357043(39.64%)	14119764(30.49%)	22644582(48.89%)	32959426(71.16%)
D2_1	48571858	43297845(89.14%)	39299964(80.91%)	3997881(8.23%)	19705604(40.57%)	19594360(40.34%)	19693854(40.55%)	19606110(40.37%)	16596591(34.17%)	22703373(46.74%)	35312346(72.7%)
D2_2	46475458	41437124(89.16%)	37598908(80.9%)	3838216(8.26%)	18839418(40.54%)	18759490(40.36%)	18842169(40.54%)	18756739(40.36%)	14963463(32.2%)	22635445(48.7%)	33705962(72.52%)
D2_3	47252628	42379743(89.69%)	38480449(81.44%)	3899294(8.25%)	19295201(40.83%)	19185248(40.6%)	19281892(40.81%)	19198557(40.63%)	15262165(32.3%)	23218284(49.14%)	34831430(73.71%)
D3_1	48092412	42759667(88.91%)	38756961(80.59%)	4002706(8.32%)	19422908(40.39%)	19334053(40.2%)	19408649(40.36%)	19348312(40.23%)	15595903(32.43%)	23161058(48.16%)	34995676(72.77%)
D3_2	42364516	38125711(89.99%)	34768342(82.07%)	3357369(7.92%)	17448068(41.19%)	17320274(40.88%)	17405740(41.09%)	17362602(40.98%)	13897482(32.8%)	20870860(49.26%)	31733198(74.91%)
D3_3	45733968	40923715(89.48%)	37053468(81.02%)	3870247(8.46%)	18579731(40.63%)	18473737(40.39%)	18557190(40.58%)	18496278(40.44%)	15202064(33.24%)	21851404(47.78%)	33721364(73.73%)
D4_1	47458658	41944652(88.38%)	38183569(80.46%)	3761083(7.92%)	19130334(40.31%)	19053235(40.15%)	19130601(40.31%)	19052968(40.15%)	15163661(31.95%)	23019908(48.51%)	34128250(71.91%)
D4_2	43374420	38521087(88.81%)	34978490(80.64%)	3542597(8.17%)	17519400(40.39%)	17459090(40.25%)	17525026(40.4%)	17453464(40.24%)	13792621(31.8%)	21185869(48.84%)	31330044(72.23%)
D4_3	55470416	49198337(88.69%)	44715019(80.61%)	4483318(8.08%)	22419714(40.42%)	22295305(40.19%)	22379889(40.35%)	22335130(40.26%)	17036065(30.71%)	27678954(49.9%)	39930136(71.98%)
CK1_1	44639390	39349482(88.15%)	30761358(68.91%)	8588124(19.24%)	15417846(34.54%)	15343512(34.37%)	15422723(34.55%)	15338635(34.36%)	12360162(27.69%)	18401196(41.22%)	27348128(61.26%)
CK1_2	43642362	37154859(85.13%)	33208150(76.09%)	3946709(9.04%)	16622474(38.09%)	16585676(38.0%)	16653633(38.16%)	16554517(37.93%)	13170654(30.18%)	20037496(45.91%)	29098050(66.67%)
CK1_3	50905702	45192004(88.78%)	38500514(75.63%)	6691490(13.14%)	19269815(37.85%)	19230699(37.78%)	19330390(37.97%)	19170124(37.66%)	15979187(31.39%)	22521327(44.24%)	34728476(68.22%)

CK2_1	47260316	40834469(86.4%)	33620383(71.14%)	7214086(15.26%)	16866737(35.69%)	16753646(35.45%)	16839199(35.63%)	16781184(35.51%)	14130312(29.9%)	19490071(41.24%)	29641466(62.72%)
CK2_2	46729074	41229469(88.23%)	34607357(74.06%)	6622112(14.17%)	17325648(37.08%)	17281709(36.98%)	17348437(37.13%)	17258920(36.93%)	12902577(27.61%)	21704780(46.45%)	31393510(67.18%)
CK2_3	43452028	38042698(87.55%)	30925158(71.17%)	7117540(16.38%)	15492039(35.65%)	15433119(35.52%)	15490967(35.65%)	15434191(35.52%)	12876366(29.63%)	18048792(41.54%)	27604514(63.53%)
CK3_1	42747128	36672447(85.79%)	33293051(77.88%)	3379396(7.91%)	16680384(39.02%)	16612667(38.86%)	16664011(38.98%)	16629040(38.9%)	12190194(28.52%)	21102857(49.37%)	29692836(69.46%)
CK3_2	49269064	42831177(86.93%)	35234875(71.52%)	7596302(15.42%)	17668320(35.86%)	17566555(35.65%)	17680216(35.89%)	17554659(35.63%)	14522313(29.48%)	20712562(42.04%)	30841276(62.6%)
CK3_3	47790358	41589578(87.03%)	34962432(73.16%)	6627146(13.87%)	17514661(36.65%)	17447771(36.51%)	17514724(36.65%)	17447708(36.51%)	14135549(29.58%)	20826883(43.58%)	30858776(64.57%)
CK4_1	49721182	42901316(86.28%)	36618542(73.65%)	6282774(12.64%)	18381400(36.97%)	18237142(36.68%)	18354274(36.91%)	18264268(36.73%)	15083714(30.34%)	21534828(43.31%)	32121572(64.6%)
CK4_2	45027642	39019882(86.66%)	31650586(70.29%)	7369296(16.37%)	15891809(35.29%)	15758777(35.0%)	15874935(35.26%)	15775651(35.04%)	13536301(30.06%)	18114285(40.23%)	27579276(61.25%)
CK4_3	46877574	40881595(87.21%)	34676578(73.97%)	6205017(13.24%)	17398742(37.12%)	17277836(36.86%)	17382027(37.08%)	17294551(36.89%)	14436227(30.8%)	20240351(43.18%)	30666388(65.42%)

Supplementary Table S4 Statistics of comparison between all samples and reference genome under light and dark treatment.

Sample	total_reads	total_map	unique_map	multi_map	read1_map	read2_map	positive_map	negative_map	splice_map	unsplice_map	proper_map
D1_1	45436082	39674351(87.32%)	35941482(79.1%)	3732869(8.22%)	18008578(39.63%)	17932904(39.47%)	18009745(39.64%)	17931737(39.47%)	14040441(30.9%)	21901041(48.2%)	31896038(70.2%)
D1_2	43074982	37559255(87.2%)	34069948(79.09%)	3489307(8.1%)	17069365(39.63%)	17000583(39.47%)	17065518(39.62%)	17004430(39.48%)	13105051(30.42%)	20964897(48.67%)	30405288(70.59%)
D1_3	46314858	40562616(87.58%)	36764346(79.38%)	3798270(8.2%)	18436930(39.81%)	18327416(39.57%)	18407303(39.74%)	18357043(39.64%)	14119764(30.49%)	22644582(48.89%)	32959426(71.16%)
D2_1	48571858	43297845(89.14%)	39299964(80.91%)	3997881(8.23%)	19705604(40.57%)	19594360(40.34%)	19693854(40.55%)	19606110(40.37%)	16596591(34.17%)	22703373(46.74%)	35312346(72.7%)
D2_2	46475458	41437124(89.16%)	37598908(80.9%)	3838216(8.26%)	18839418(40.54%)	18759490(40.36%)	18842169(40.54%)	18756739(40.36%)	14963463(32.2%)	22635445(48.7%)	33705962(72.52%)
D2_3	47252628	42379743(89.69%)	38480449(81.44%)	3899294(8.25%)	19295201(40.83%)	19185248(40.6%)	19281892(40.81%)	19198557(40.63%)	15262165(32.3%)	23218284(49.14%)	34831430(73.71%)
D3_1	48092412	42759667(88.91%)	38756961(80.59%)	4002706(8.32%)	19422908(40.39%)	19334053(40.2%)	19408649(40.36%)	19348312(40.23%)	15595903(32.43%)	23161058(48.16%)	34995676(72.77%)
D3_2	42364516	38125711(89.99%)	34768342(82.07%)	3357369(7.92%)	17448068(41.19%)	17320274(40.88%)	17405740(41.09%)	17362602(40.98%)	13897482(32.8%)	20870860(49.26%)	31733198(74.91%)
D3_3	45733968	40923715(89.48%)	37053468(81.02%)	3870247(8.46%)	18579731(40.63%)	18473737(40.39%)	18557190(40.58%)	18496278(40.44%)	15202064(33.24%)	21851404(47.78%)	33721364(73.73%)
D4_1	47458658	41944652(88.38%)	38183569(80.46%)	3761083(7.92%)	19130334(40.31%)	19053235(40.15%)	19130601(40.31%)	19052968(40.15%)	15163661(31.95%)	23019908(48.51%)	34128250(71.91%)
D4_2	43374420	38521087(88.81%)	34978490(80.64%)	3542597(8.17%)	17519400(40.39%)	17459090(40.25%)	17525026(40.4%)	17453464(40.24%)	13792621(31.8%)	21185869(48.84%)	31330044(72.23%)
D4_3	55470416	49198337(88.69%)	44715019(80.61%)	4483318(8.08%)	22419714(40.42%)	22295305(40.19%)	22379889(40.35%)	22335130(40.26%)	17036065(30.71%)	27678954(49.9%)	39930136(71.98%)
CK1_1	44639390	39349482(88.15%)	30761358(68.91%)	8588124(19.24%)	15417846(34.54%)	15343512(34.37%)	15422723(34.55%)	15338635(34.36%)	12360162(27.69%)	18401196(41.22%)	27348128(61.26%)
CK1_2	43642362	37154859(85.13%)	33208150(76.09%)	3946709(9.04%)	16622474(38.09%)	16585676(38.0%)	16653633(38.16%)	16554517(37.93%)	13170654(30.18%)	20037496(45.91%)	29098050(66.67%)
CK1_3	50905702	45192004(88.78%)	38500514(75.63%)	6691490(13.14%)	19269815(37.85%)	19230699(37.78%)	19330390(37.97%)	19170124(37.66%)	15979187(31.39%)	22521327(44.24%)	34728476(68.22%)

CK2_1	47260316	40834469(86.4%)	33620383(71.14%)	7214086(15.26%)	16866737(35.69%)	16753646(35.45%)	16839199(35.63%)	16781184(35.51%)	14130312(29.9%)	19490071(41.24%)	29641466(62.72%)
CK2_2	46729074	41229469(88.23%)	34607357(74.06%)	6622112(14.17%)	17325648(37.08%)	17281709(36.98%)	17348437(37.13%)	17258920(36.93%)	12902577(27.61%)	21704780(46.45%)	31393510(67.18%)
CK2_3	43452028	38042698(87.55%)	30925158(71.17%)	7117540(16.38%)	15492039(35.65%)	15433119(35.52%)	15490967(35.65%)	15434191(35.52%)	12876366(29.63%)	18048792(41.54%)	27604514(63.53%)
CK3_1	42747128	36672447(85.79%)	33293051(77.88%)	3379396(7.91%)	16680384(39.02%)	16612667(38.86%)	16664011(38.98%)	16629040(38.9%)	12190194(28.52%)	21102857(49.37%)	29692836(69.46%)
CK3_2	49269064	42831177(86.93%)	35234875(71.52%)	7596302(15.42%)	17668320(35.86%)	17566555(35.65%)	17680216(35.89%)	17554659(35.63%)	14522313(29.48%)	20712562(42.04%)	30841276(62.6%)
CK3_3	47790358	41589578(87.03%)	34962432(73.16%)	6627146(13.87%)	17514661(36.65%)	17447771(36.51%)	17514724(36.65%)	17447708(36.51%)	14135549(29.58%)	20826883(43.58%)	30858776(64.57%)
CK4_1	49721182	42901316(86.28%)	36618542(73.65%)	6282774(12.64%)	18381400(36.97%)	18237142(36.68%)	18354274(36.91%)	18264268(36.73%)	15083714(30.34%)	21534828(43.31%)	32121572(64.6%)
CK4_2	45027642	39019882(86.66%)	31650586(70.29%)	7369296(16.37%)	15891809(35.29%)	15758777(35.0%)	15874935(35.26%)	15775651(35.04%)	13536301(30.06%)	18114285(40.23%)	27579276(61.25%)
CK4_3	46877574	40881595(87.21%)	34676578(73.97%)	6205017(13.24%)	17398742(37.12%)	17277836(36.86%)	17382027(37.08%)	17294551(36.89%)	14436227(30.8%)	20240351(43.18%)	30666388(65.42%)

Supplementary Table S5 Primers used in this study

Genes	Primer
UBC-F	TGTGTGTGTGTGTGTGTGTCC
UBC-R	CCTTTCTCCACGGTCTTCAA
LcACOT1-F	CTGCTGTTTTCGAACGAGGT
LcACOT1-R	GAGCATCCCTTGATAGAAGATTG
LcHMGS1-F	GTCGCAGCAGAAGGATGTC
LcHMGS1-R	TTGCACCATCATGAGCTTCC
LcDXS3-F	TGTCTCCCATGTACAGACTTGC
LcDXS3-R	AACAACCTTCCCCTCATCATC
LcDXR-F	AGAGCTAGTGACCAGTCCGT
LcDXR-R	AAGCCAGGACGGGTCTTTTC
LcMDS-F	ACACCGACCCCAAATGGA
LcMDS-R	TGAGCCTAACCGCTTCTTTC
LcTPS19-F	GTCTATCCAGTGTTACATGTATGAAGC
LcTPS19-R	TTGAAGAAAGGGAGTGAAGTAAACT
